# Acute and chronic animal models for the evaluation of anti-diabetic agents

**DOI:** 10.1186/1475-2840-11-9

**Published:** 2012-01-19

**Authors:** Suresh Kumar, Rajeshwar Singh, Neeru Vasudeva, Sunil Sharma

**Affiliations:** 1 Pharmacology Division, Department of Pharmaceutical Sciences, Guru Jambheshwar University of Science and Technology, Post Box: 38, Hisar-125001, India

**Keywords:** Streptozotocin, alloxan, diabetic rats, animal models, diabetes

## Abstract

Diabetes mellitus is a potentially morbid condition with high prevalence worldwide thus being a major medical concern. Experimental induction of diabetes mellitus in animal models is essential for the advancement of our knowledge and understanding of the various aspects of its pathogenesis and ultimately finding new therapies and cure. Experimental diabetes mellitus is generally induced in laboratory animals by several methods that include: chemical, surgical and genetic (immunological) manipulations. Most of the experiments in diabetes are carried out in rodents, although some studies are still performed in larger animals. The present review highlights the various methods of inducing diabetes in experimental animals in order to test the newer drugs for their anti-diabetic potential.

## Introduction

WHO reports Diabetes mellitus as one of the most common public health problems which will affect a total population of 220 million worldwide in the year 2020 [1,2]. The increasing prevalence of diabetes mellitus worldwide is a major societal issue because diabetes is a complex and multifactorial origin disease.

The prevalence of diabetes is rising all over the world due to population growth, aging, urbanization and an increase of obesity and physical inactivity. Unlike in the West, where older persons are most affected, diabetes in Asian countries is disproportionately high in young to middle-aged adults. This could have long-lasting adverse effects on a nation's health and economy, especially for developing countries. The International Diabetes Federation (IDF) estimates the total number of people in India with diabetes to be around 50.8 million in 2010, rising to 87.0 million by 2030.

According to recent estimates, approximately 285 million people worldwide (6.6%) in the 20-79 year age group will have diabetes in 2010 and by 2030, 438 million people (7.8%) of the adult population, is expected to have diabetes [3]. The largest increases will take place in the regions dominated by developing economies. According to the World Health Organization (WHO) criteria, the prevalence of known diabetes was 5.6% and 2.7% among urban and rural areas, respectively [4].

Diabetes mellitus, often simply referred to as diabetes--is a group of metabolic diseases in which a person has high blood sugar, either because the body does not produce enough insulin, or because cells do not respond to the insulin that is produced. This high blood sugar produces the classical symptoms of polyuria (frequent urination), polydipsia (increased thirst) and polyphagia (increased hunger). Diabetes mellitus can be of different types based on the cause of the disease.

## Types of diabetes

There are three main types of diabetes which are briefly described as below:

### Type 1 diabetes

It results from the body's failure to produce insulin, and presently requires the person to inject insulin. (It is also referred to as insulin-dependent diabetes mellitus, IDDM for short, and juvenile diabetes). The majority of type 1 diabetes is of the immune-mediated nature, where beta cell loss is a T-cell mediated autoimmune attack [5].

### Type 2 diabetes

It results from insulin resistance, a condition in which cells fail to use insulin properly, sometimes combined with an absolute insulin deficiency. It is also known as non-insulin-dependent diabetes mellitus, NIDDM for short, and adult-onset diabetes.

### Gestational diabetes

This type of diabetes occurs in pregnant women, who never had diabetes before, but have a high blood glucose level during pregnancy. It may precede development of type 2 diabetes mellitus [6].

### Other diabetes

Other forms of diabetes mellitus include congenital diabetes, which is due to genetic defects of insulin secretion, cystic fibrosis-related diabetes, steroid diabetes induced by high doses of glucocorticoids, and several forms of monogenic diabetes. Pre-diabetes indicate a condition that occurs when a person's blood glucose levels are higher than normal but not high enough for a diagnosis of type 2 diabetes. Many people destined to develop type 2 diabetes spend many years in a state of pre-diabetes which has been termed "America's largest healthcare epidemic [7]. Latent autoimmune diabetes of adults is a condition in which Type 1 diabetes develops in adults. Adults with LADA are frequently initially misdiagnosed as having Type 2 diabetes, based on age rather than etiology [8].

Genetic mutations (autosomal or mitochondrial) can lead to defects in beta cell function. Abnormal insulin action can be genetically determined in some cases. Any disease that causes extensive damage to the pancreas may lead to diabetes (for example chronic pancreatitis and cystic fibrosis). Diseases associated with excessive secretion of insulin antagonistic hormones can cause diabetes (which is typically resolved once the hormone excess is removed). Many drugs impair insulin secretion and some toxins damage pancreatic beta cells [9].

Experimental induction of diabetes mellitus in animal models is essential for the advancement of our knowledge and understanding of the various aspects of its pathogenesis and ultimately finding new therapies and cure. Experimental studies in animals have the great advantage to eliminate factors such as ethnicity, economic and geographic variables, drug interactions, diet, gender and age differences that importantly limit clinical studies. Indeed, appropriate animal models have provided important information on genetic and environmental risks of diabetes, and helped to dissect molecular mechanisms underlying the development, progression and therapeutic control of this disease [10]. Several methods have been used to induce diabetes mellitus in laboratory animals with variable success and many difficulties. Surgical removal of the pancreas is an effective method, however, to induce diabetes, at least 90-95% of the pancreas has to be removed [11].

The majority of studies published in the field of ethno-pharmacology between 1996 and 2006 employed chemical induced model. Streptozotocin (STZ) 69% and alloxan 31% are by far the most frequently used drugs and this model has been useful for the study of multiple aspects of the disease [12]. Both drugs exert their diabetogenic action when they are administered parenterally (intravenously, intraperitoneally or subcutaneously). The dose of these agents required for inducing diabetes depends on the animal species, route of administration and nutritional status [13].

STZ (*N*-nitro derivative of glucosamine) is a naturally occurring, broad spectrum antibiotic and cytotoxic chemical that is particularly toxic to the pancreatic, insulin producing beta cells in mammals [14-17]. Induction of experimental diabetes in the rat using STZ is very convenient and simple to use [18,19]. STZ injection leads to the degeneration of the Langerhans islets beta cells [20,21].

Alloxan, a well- known diabetogenic agent is widely used to induce type 2 diabetes in animals [22]. The drug and its reduction product dialuric acid establish a redox cycle with the formation of superoxide radicals. These radicals undergo dismutation to hydrogen peroxide. Thereafter, highly reactive hydroxyl radicals are formed by fenton reaction. The action of reactive oxygen species with a simultaneous massive increase in cytosolic calcium concentration causes rapid destruction of β cells [23]. Alloxan induced diabetes mellitus serve as a pathological biomodel for testing a substance with supposed antioxidant activities *in vivo *[24]. One of the targets of the reactive oxygen species is DNA of pancreatic islets. Its fragmentation takes place in β cells exposed to alloxan [25]. The increase in oxygen free radicals in diabetic conditions is mainly because of the effect of the diabetogenic agent alloxan.

Although the use of chemical (alloxan and STZ) to induce type 2 diabetes mellitus is the most widely used method, recently it has been widely criticized as being unsuitable because the chemical seems to destroy the β cells and create more of type 1 than type 2 diabetes [26].

## Diagnosis of diabetes [27]

Different current diagnostic tests for diabetes mellitus are,

### (A) Measurement of blood glucose

The current WHO diagnostic criteria for diabetes are measurement of blood glucose level. The fasting plasma glucose greater than 7.0 mmol/l (126 mg/dl) or 2-h plasma glucose greater than 11.1 mmol/l (200 mg/dl) indicate that the person may have diabetes. Glucose should be measured immediately after collection by near patient testing, or if a blood sample is collected, plasma should be immediately separated, or the sample should be collected into a container with glycolytic inhibitors and placed on ice-water until separated prior to analysis.

### (B) Oral glucose tolerance test (OGTT)

OGTT is used as a diagnostic test as fasting plasma glucose alone fails to diagnose approximately 30% of cases.

### (C) Glycated Haemoglobin (HbA1c)

HbA1c can be used as a diagnostic test for diabetes providing that stringent quality assurance tests are in place and assays are standardized to criteria aligned to the international reference values, and there are no conditions present which preclude its accurate measurement. An HbA1c of 6.5% is recommended as the cut point for diagnosing diabetes. A value of less than 6.5% does not exclude diabetes diagnosed using glucose tests. Currently HbA1c is not considered a suitable diagnostic test for diabetes or intermediate hyperglycemia [28].

### (D) Fasting blood glucose test

Blood glucose levels are checked after fasting for between 12 and 14 hours. Water can be taken during this time, but should strictly avoid any other beverage. Patients with diabetes may be asked to delay their diabetes medication or insulin dose until the test is completed [27].

### (E) Random blood glucose test

Blood glucose levels are checked at various times during the day, and it doesn't matter the time of eating. Blood glucose levels tend to stay constant in a person who doesn't have diabetes [27].

## Cardiovascular complications associated with diabetes

Several cardiovascular complications have been associated with diabetes. These include Coronary Artery Disease, Cerebral Vascular Disease, Peripheral Arterial Disease, Stroke, TIAs, Heart Failure, Peripheral Arterial Disease, High blood pressure etc. The relative risks for total cardiovascular disease, coronary heart disease, stroke, other cardiovascular disease, and all-cause mortality increase two- to threefold in diabetic patients, even after adjustment for several other known cardiovascular disease risk factors.

An association between overt diabetes and cardiovascular disease has been observed in many studies [29,30]. In the developed world, the risk for cardiovascular disease has increased two- to four fold among diabetic patients compared with non diabetic persons within their corresponding population. Among persons with NIDDM in the United States, up to 75% of deaths are attributed to ischemic heart disease or other heart and vascular disease [31]. Although the cardiovascular disease mortality rate has recently decreased in diabetic persons as well as in normoglycemic persons, a higher risk for cardiovascular disease remains a major problem for diabetic patients [32].

### Coronary Artery Disease

Coronary artery disease, also called ischemic heart disease, is caused by a hardening or thickening of the walls of the blood vessels that go to your heart. Blood supplies oxygen and other materials needed by heart for normal functioning. If the blood vessels to heart are narrowed or blocked by fatty deposits which normally is reported to increase in a diabetic person the blood supply is reduced or cut off, resulting in a heart attack.

### Cerebral Vascular Disease

Cerebral vascular disease affects blood flow to the brain, leading to strokes and TIAs. It is caused by narrowing, blocking, or hardening of the blood vessels that go to the brain or by high blood pressure.

### Peripheral Arterial Disease

Narrowing or blockage of the blood vessels in the legs, a condition called peripheral arterial disease, can also occur in people with diabetes.

### Stroke

A stroke results when the blood supply to the brain is suddenly cut off, which can occur when a blood vessel in the brain or neck is blocked or the vessel bursts. Brain cells are then deprived of oxygen and die. A stroke can result in problems with speech or vision or can cause weakness or paralysis. Most strokes are caused by fatty deposits or blood clots jelly-like clumps of blood cells that narrow or block one of the blood vessels in the brain or neck. A blood clot may stay where it formed or can travel within the body. People with diabetes are at increased risk for strokes caused by blood clots. A stroke may also be caused by a bleeding blood vessel in the brain. Called an aneurysm, a break in a blood vessel can occur as a result of high blood pressure or a weak spot in a blood vessel wall.

### Transient Ischemic Attacks (TIAs)

TIAs are caused by a temporary blockage of a blood vessel to the brain. This blockage leads to a brief, sudden change in brain function, such as temporary numbness or weakness on one side of the body. Sudden changes in brain function also can lead to loss of balance, confusion, blindness in one or both eyes, double vision, difficulty speaking, or a severe headache. However, most symptoms disappear quickly and permanent damage is unlikely. If symptoms do not resolve in a few minutes, rather than a TIA, the event could be a stroke. The occurrence of a TIA means that a person is at risk for a stroke sometime in the future.

### Heart Failure

Heart failure is a chronic condition in which the heart cannot pump blood properly. Heart failure develops over a period of years, and symptoms can get worse over time. People with diabetes have at least twice the risk of heart failure as other people. Blockage of the blood vessels and high blood glucose levels also can damage heart muscle and cause irregular heartbeats. People damaged heart muscle (a condition called cardiomyopathy) may have no symptoms in the early stages, but later they may experience weakness, shortness of breath, a severe cough, fatigue, and swelling of the legs and feet. Diabetes can also interfere with pain signals normally carried by the nerves, explaining why a person with diabetes may not experience the typical warning signs of a heart attack.

### Peripheral Arterial Disease

Another condition related to heart disease and common in people with diabetes is peripheral arterial disease (PAD). With this condition, the blood vessels in the legs are narrowed or blocked by fatty deposits, decreasing blood flow to the legs and feet. PAD increases the chances of a heart attack or stroke occurring. Poor circulation in the legs and feet also raises the risk of amputation. Sometimes people with PAD develop pain in the calf or other parts of the leg when walking, which is relieved by resting for a few minutes.

## 2. Experimental Models

Experimental diabetes mellitus is generally induced in laboratory animals by several methods that include: chemical, surgical and genetic (immunological) manipulations. Most of the experiments in diabetes are carried out in rodents, although some studies are still performed in larger animals.

The animal models employed for screening anti-diabetic agents can be broadly classified into three types which are enlisted below [33].

**A **Methods to induce experimental diabetes mellitus

**B **Genetically diabetic animals

**C **Miscellaneous Models

A. Methods to induce experimental diabetes mellitus can be further sub-classified as under:-

a) Pancreatectomy in dogs

b) Alloxan induced diabetes

c) Streptozotocin induced diabetes

d) Hormone induced diabetes

e) Virus induced diabetes

f) Other diabetogenic compounds

g) Insulin deficiency due to insulin antibodies

B. Genetically diabetic animals can be further sub-classified as under:

a) Spontaneously diabetic rats

b) Spontaneously diabetic mice

c) Chinese hamsters

d) Other species with inherited diabetic symptoms

e) Transgenic animals

C. Miscellaneous Models

a) Invertebrate animal model

b) Diet Induced metabolic dysregulation

### Pancreatectomy in dogs

#### General Considerations

Polyuria, polydipsia, polyphagia, and severe glucosuria take place after removal of the pancreas in dogs. The reduction of the elevated blood sugar levels in pancreatectomized dogs by injection of extracts of the pancreatic glands further proved the existence of hormone in pancreas [34]. The technique of complete pancreatectomy in the dog was first described in detail by Foà [35] and then by Sirek [36] and there after that it has been extensively used by various scientists as an animal model for diabetes mellitus

#### Method

Male Beagle dogs weighing 12-16 kg are anesthetized with an intravenous injection of 50 mg/kg pentobarbital sodium and placed on its back. A midline incision is made from the xyphoid process reaching well below the umbilicus. Bleeding vessels are ligated and the abdomen is entered through the linea alba. The falciform ligament is carefully removed and the vessels are ligated. A self-retaining retractor is applied. By passing the right hand along the stomach to the pylorus, the duodenum with the head of the pancreas is brought into the operating field. First, the mesentery at the unicate process is cut and the process itself is dissected free. The glandular tissue is peeled off from the inferior pancreatico-duodenal artery and vein. The vessels themselves are carefully preserved along a line of cleavage which exists between the pancreas, the pancreatico-duodenal vessels and the duodenal wall, the pancreas is separated from the duodenum and from the carefully preserved pancreatico-duodenal vessels. The small vessels to the pancreas are ligated. The dissection is carried out from both sides of the duodenum. In the area of the accessory pancreatic duct, the glandular tissue being attached very firmly has to be carefully removed in order to leave no residual pancreatic tissue behind. The pancreatic duct is cleaned, doubly ligated and cut between the ligatures. The dissection proceeds until one encounters a small lobe containing the main pancreatic duct. The glandular tissue adheres here firmly to the duodenum. Blunt dissection and ligation of the vessels is followed by ligation of the pancreatic duct. By pulling on the pylorus and the stomach, the pyloric and the splenic parts of the pancreas are delivered into the wound. The duodenal part is placed back into the abdominal cavity. The mesentery of the body and tail of the pancreas is cut with scissors. The small vessels are doubly ligated and cut. The pancreatic tissue is bluntly dissected from the splenic vessels. The pancreatic branches of the splenic vessels are doubly ligated and cut. Working in direction from the spleen to the pylorus, the pyloric part of the pancreas is the last one to be dissected. Finally, all pancreatic tissue is removed. The surgical field is checked once more for pancreatic remnants. The concavity of the duodenum and its mesentery is approximated by a few silk stitches and the omentum is wrapped around the duodenum. Retroperitoneal injection of 5 ml 1% procaine solution is given to prevent intussusception of the gut. 250000 IU penicillin G in saline solution is instilled into the peritoneal cavity. The abdominal wall and the subcutaneous layer are closed by sutures and finally the skin is sutured with continuous everting mattress stitches. After the operation, the animal receives via a jugular vein catheter for 3-4 days the following treatment: 1000 ml 10% glucose solution with 10 IU human insulin, 3 ml 24% sulfadioxin/trimethoprim solution, 2 ml 50% metamizol and 400 IU secretin. On the third day, the animal is offered milk. After the animal has passed the first milk feces, it is given daily dry food together with a preparation of pancreatic enzymes. Insulin is substituted with a single daily subcutaneous dose of 34 IU Retard-Insulin. Vitamin D3 is given every three months as a intramuscular injection of 1 ml.

### Alloxan induced diabetes

#### Objective

Surveys on chemically induced diabetes in animals were given by Frerichs and Creutzfeldt [37]. Alloxan administration cause hyperglycemia and glucosuria has been described in several species, such as in dogs [38], in rabbits [39], in rats [40] and in other species [41]. Alloxan resistance has been found in Guinea pig [42]. Dosage and treatment regimen have been elaborated for the most frequently used species. In most species a triphasic time course is observed: an initial rise of glucose is followed by a decrease, probably due to depletion of islets from insulin, again followed by a sustained increase of blood glucose. Alloxan generally produces greater cytotoxicity owing to its conversion to anionic radicals.

#### Method

Rabbits weighing 2.0 to 3.5 kg are infused with 150 mg/kg alloxan monohydrate (5.0 g/100 ml, pH 4.5) through ear vein for 10 min. This results in occurrence of hyperglycemia and uricosuria in 70% of the animals. The rest of the animals either die or are only temporarily hyperglycemic [43]. Rats of Wistar or Sprague-Dawley strain weighing 150-200 g are injected subcutaneously with 100-175 mg/kg alloxan [44]. Male Beagle dogs weighing 15-20 kg are injected intravenously with 60 mg/kg alloxan. Subsequently, the animals receive daily 1 000 ml 5% glucose solution with 10 IU Regular insulin for one week and canned food ad libitum. Thereafter, a single daily dose of 28 IU insulin is administered subcutaneously [45]. The method was modified in which rats of 2, 4 and 6 days were injected with 200 mg/kg of alloxan monohydrate after a 16 h fast [46].

### Streptozotocin induced diabetes

#### Objective

Rakieten et al reported the diabetogenic activity of the antibiotic Streptozotocin [47]. STZ is cytotoxic especially to beta- cells of the pancreas. Diabetes induction in laboratory animals, mostly in rats, by STZ has become a valuable tool in diabetes research being used by many investigators. Parp-deficient mice are almost resistant to STZ-induced diabetes [48].

#### Method

Male Wistar rats weighing 150-220 g are used. STZ (60 mg/kg) is injected intravenously. The serum insulin values decrease up to 4 times, after six to eight hours of injection, resulting in a hypoglycemic phase after persistent hyperglycemia. Diabetic symptoms severity and onset depend on the dose of STZ. After the dose of 60 mg/kg i.v., symptoms occur already after 24-48 h with hyperglycemia up to 800 mg%, glucosuria and ketonemia. Histologically degranulation of the beta cells is seen. After 10-14 days a steady state is reached allowing using the animals for pharmacological tests.

### Hormone induced diabetes

#### Growth hormone induced diabetes

The diabetogenic action of pure anterior pituitary growth hormone in cats has already been described [49]. The repeated administration of growth hormone in adult dogs and cats induce intensive diabetes with all symptoms including severe ketonuria and ketonemia while rats do not show any sign of diabetes on a similar treatment, but grow faster [50].

#### Corticosteroid induced diabetes

Cortisone can induce hyperglycemia and glucosuria in treated rats [51]. In the guinea pig and in the rabbit, experimental corticoid diabetes could be obtained without forced feeding [52]. In the rat, the adrenal cortex, stimulated by corticotrophin has the capacity to secrete amounts of steroids which induce steroid diabetes [53].

### Virus induced diabetes

Virus infections and beta-cell specific autoimmunity may induce Juvenile-onset (type I) diabetes mellitus [54]. Infection and destruction of pancreatic beta-cell by D-variant of encephalomyocarditis virus (EMC-D) induce diabetes [55]. Susceptibility to cause insulin-dependent diabetes in mouse strains is similar to human. Adult, male ICR Swiss mice are susceptible to the diabetogenic effect of the D-variant of encephalomyocarditis virus present in ICR Swiss mice but C3H/HeJ male mice are resistant. The severity and incidence of diabetes is increased if the animals are pretreated with cyclosporine (A potent immunosuppressive drug).

### Other diabetogenic compounds

#### Objective

Symptoms of diabetes and obesity induction have been found by several other compounds such as dithizone [56] or goldthioglucose [57] or monosodium glutamate [58]. Histopathology reveals complete or partial degranulation of beta cells by the action of dithiazone.

#### Method

Goldberg et al used various chelators, such as dithizone, 8-(p-toluene-sulfonylamino)-quinoline (8-TSQ), and 8-(benzenesulfanylamino)-quinoline for induction of diabetes in experimental animals [59]. Dithizone is injected to cats, rabbits, golden hamsters and mice in a single i.v. dose of 40-100 mg/kg. In rabbits dithizone injection causes a triphasic glycemic reaction. After 2 h initial hyperglycemia is detected followed by a normoglycemic phase after 8 h and after 24-72 h a permanent hyperglycemia.

### Insulin deficiency due to insulin antibodies

#### Objective

Guinea pig anti-insulin serum can induce a transient diabetic syndrome [60]. Guinea pig anti-insulin serum induces unique effect. This is due to neutralization by insulin antibodies of endogenous insulin secreted by the injected animal. This induces a state of insulin deficiency. Ketonemia, ketonuria, glucosuria, and acidosis are induced by larger dose. The diabetic syndrome is reversible at lower doses after a few hours.

#### Method

Male guinea pigs weighing 300-400 g are used. 1 mg dose of Bovine insulin is injected subcutaneously in divided dose. Injections are given at monthly intervals. 10 ml blood from every animal once a month is withdrawn by cardiac puncture. 0.25-1.0 ml guinea pig anti-insulin serum is injected i.v. to rats. Anti-insulin serum induces a dose-dependent increase in blood glucose up to 300 mg%.

## Genetically diabetic animals

### General considerations

Rodents have been described to exhibit spontaneous diabetes mellitus on a hereditary basis. Since the discovery of leptin [61] and its downstream signal transduction cascade [62], new insight of the genetics of diabetic and obese animal disease models were derived. Many genetically diabetic animal models exhibit defects in the leptin pathway, various mutations in the mouse resulted in leptin deficiency. Biologically inactive carboxipeptidase E are formed as a result of fat mutation in the mouse, which processes the prohormone conversion of POMC into α-MSH, and activates the hypothalamic MC4 receptor. Ubiquitous expression of the Agouti protein was exhibited by yellow mouse, which represents an antagonist of the hypothalamic MC4 receptor. Symptoms of diabetes and obesity are overlapping in many animal models

### Critical assessment

The pathophysiological mechanisms which finally lead to the diabetes phenotype (hyperglycemia, hyperinsulinemia and insulin resistance) exhibited by the various animal disease models for non-insulin dependent diabetes do not necessarily be identical to those in human disease. Therefore, detailed knowledge about the patho-physiology of these animal disease models is a prerequisite for interpretation of experimental results and their value for the human disease.

### Spontaneously diabetic rats

In several strains of rats occurrence of spontaneous diabetes has been reported:

#### Bio Breeding (BB) Rat

The BB rat is a model of spontaneous diabetes [63]. It is associated with insulin deficiency and insulitis due to autoimmune destruction of pancreatic beta cells. The onset of clinical diabetes generally occurs at 60-120 days of age. Severe hyperglycemia persists after several days associated with hypoinsulinemia and ketosis. Immunosuppressive agent mycophenolate mofetil can prevent the development of diabetes in BB rats [64]. The features of a subline of diabetes-prone BB rats were characterized by Kloting and Vogt [65].

#### WBN/KOB rat

These animals of Wistar strain, named as WBN/Kob rat exhibit impaired glucose tolerance and glucosuria at 21 weeks of age. Decrease in the number and size of islets after 12 weeks of age is observed. Yagihashi observed fibrinous exudation and degeneration of pancreatic tissue in the exocrine part [66]. In 16 weeks old male rat degeneration takes place mainly around islets and pancreatic ducts. These rats develop demyelination, predominantly motor neuropathy.

#### Cohen diabetic rat

Cohen described that hyperglycemia, glucosuria and hyperinsulinemia are characteristics of Cohen rats [67]. Late development of hypoinsulinemia, insulin resistance and a decrease in the number and sensitivity of insulin receptors in Cohen rats is observed. Diabetes related complications are develop in rats when fed a diet rich in sucrose or other refined sugars, but not when fed a starch or stock diet [68].

#### Goto-Kakizaki (GK) rat

GK rats are non-obese, insulin-resistant. GK rats are highly inherited strain of Wistar rats that spontaneously develop type II diabetes. 2 to 4 weeks after birth defects in glucose stimulated insulin secretion, peripheral insulin resistance, and hyperinsulinemia are seen along with impaired skeletal muscle glycogen synthase activation by insulin. It is accompanied by chronic activation of diacylglycerol-sensitive protein kinase C [69].

#### Zucker-fatty rat

Zucker described a classic model of hyperinsulinemic obesity. Obesity occurs due to a simple autosomal recessive (*fa*) gene at an early age [70]. Obese rats manifest peripheral insulin resistance similar to human NIDDM [71]. However, throughout life their blood sugar level is usually normal.

#### Zucker diabetic fatty rat (ZDF/DRT-FA)

Peterson derived the obese Zucker Diabetic Fatty rat originally from the Zucker fatty rat. This strain suffers with hyperglycemia about 20 mmol/l [72]. The males and females of 6 to 8 weeks and 9 to 11 weeks become diabetic respectively. Lee described that lipotoxicity to the β-cell is the cause of diabetes [73]. Extreme hyperphagia due to the loss of calories by glucosuria and obesity besides hyperglycemia are the characteristics of these animals.

#### WDF/TA-FA rat

Wistar fatty rats are genetically obese, hyperglycemic. Velasquez et al. developed Wistar Kyoto rat by the transfer of the fatty (*fa*) gene from the Zucker rat [74]. The Wistar fatty rat exhibits obesity, hyperinsulinemia, hyperlipidemia, and hyperphagia, however they are more insulin resistant. In females hyperglycemia is not observed usually but it can be induced by addition of sucrose to the diet.

#### OLETF rat

In 1984 diabetic rat with polyuria, polydipsia, and mild obesity was discovered in an out bred colony of Long-Evans rats. A strain of rats was developed from this rat by selective breeding. Characteristic features of OLETF rats are: (1) hyperglycemia onset is late (after 18 weeks of age), (2) a chronic course of disease, (3) mild obesity, (4) inheritance by males, (5) hyperplastic foci of pancreatic islets, and (6) renal complications (nodular lesions). Human NIDDM features resemble with the clinical and pathological features of disease in OLETF rats. Aizawa et al. (1995) found that OLETF rats from the age of 4 to 12 weeks completely prevented the development of obesity and insulin resistance when diazoxide (0.2% in diet), an inhibitor of insulin secretion is administred. Insulin resistance preceded impaired insulin secretion in OLETF rats [75].

#### ESS-rat

Tarrés et al reported that the occurrence of spontaneous diabetes in a colony of rats (Stilman Saldago), called them as ESS-rat [76]. From the age of 2 months onwards the animals show abnormal glucose tolerance tests. The syndrome consists of a mild type of diabetes that does not diminish the longevity of the animals. Dumm et al. showed the disruption of the islet architecture and fibrosis of the stroma in six months old rats [77].

#### OBESE SHR rat

Koletsky developed the strain of obese SHR rats [78]. This strain is produced by mating a spontaneous hypertensive female rat of the Kyoto-Wistar strain with a normotensive Sprague- Dawley male. Substrains such as the JCRLA-corpulent rat were developed from these rats. Russell et al described that these rats exhibits a syndrome characterized by obesity, hypertriglyceridemia and hyperinsulinemia [79]. Friedman et al found in the obese spontaneously hypertensive Koletsky rat that the insulin receptor signaling was reduced [80].

#### SHR/N-CP rat

Adamo et al developed the congenic SHR/N-cp rat strain at the National Institutes of Health, USA [81]. This strain was derived by mating a male Koletsky rat heterozygous for the corpulent gene (cp/+) to a female rat of the Okamoto strain. These rats exhibit obesity, mild hypertension, hyperinsulinemia, and glucose intolerance.

#### BHE rat

The BHE rat colony was originally developed by breeding black and white hooded rats of the Pennsylvania State College strain and albino rats of the Yale (Osborne Mendell) strain. The BHE rats exhibited the diabetic state only at maturity. These rats have hyperinsulinemia at 50 days of age. Hyperglycemia is associated with glucose intolerance and tissue resistance to insulin [82].

### Spontaneously diabetic mice

#### KK mouse

Nakamura reported a diabetic strain of the KK-mouse. The animals were moderately obese and showed polyphagia and polyuria [83]. Mice at the age of seven months or older showed glucosuria and blood sugar levels up to 320 mg%. The pancreatic insulin content was increased, but histologically degranulation of the beta-cells and hypertrophy of the islets were found. Sections of the liver showed a reduction of glycogen and an increase in lipid content.

#### KK-AY mouse

Iwatsuka et al reported the mice carrying the yellow obese gene (Ay) [84]. In these mice blood glucose and circulating insulin levels as well as HbA1c levels are increased progressively from 5 weeks of age. Degranulation and glycogen infiltration of beta-cells and lipogenesis by liver and adipose tissue were increased. Diani et al reported that the renal involvement is uniquely marked by early onset and rapid development of glomerular basement membrane thickening [85]. The extrapancreatic action of antidiabetic drugs, such as glimepiride, a novel sulfonylurea can be demonstrated by using KK-Ay mice.

#### NOD mouse

This strain was established by inbreeding diabetic CTS mice derived originally from the JCLICR strain. The NOD mouse is a model of insulin dependent diabetes mellitus and develops hypoinsulinemia secondary to autoimmune destruction of pancreatic β cells. Between 100 to 200 days of age NOD mice develop diabetes. The NOD mouse does not survive for more than one month if insulin treatment is not given. They usually die from ketosis. Baeder et al, reported that an immunomodulating drug [86] or a soluble interleukin-1 receptor [87] use can prevent the onset of diabetes. Verdaguer et al observed that the insulin-dependent diabetes mellitus in NOD mice is the result of a CD4+ and CD8+ T cell-dependent auto-immune process directed against the pancreatic β-cells [88].

#### OBESE hyperglycemic mice

Bleisch et al observed hereditary diabetes in genetically obese mice. These mice are glycosuric, the non-fasting blood sugar levels are about 300 mg%, but neither ketonuria nor coma is observed [89]. One of the most interesting features is insulin resistance. The islands of Langerhans are hypertrophic, their insulin content is increased. No pathological changes shown in kidneys and other organs. Obviously, the diabetic condition of the human diabetic patient is different from that of obese hyperglycemic mice. Halaas et al, reported the substitution of leptin reverses the obese and diabetic phenotype completely [90]. Herberg and Coleman described the other strains or substrains of mice with obesity and hyperglycemia [91].

#### Diabetes mouse (*DB/DB*)

Autosomal recessive mutation derived diabetes in *db/db *mouse strain is spontaneously diabetic mice which was identified as a mutation in the leptin receptor gene [92]. A severe diabetic syndrome is developed in the mice characterized by early onset of hyperinsulinemia, and hyperglycemia [93]. They also developed significant nephropathy. Mutations on the leptin receptor result in an obese phenotype identical to that of *ob *mice. C57BL/KsJ *ob/ob *mice are phenotypically the same as other strains of *db *mice. The leptin receptor (Ob-R) gene encodes 5 alternatively spliced forms, Ob-Ra, Ob-Rb, Ob-Rc, Ob-Rd. In the C57BL/KsJ *ob/ob *mouse strain, the Ob-Rb transcript contains an insert with a premature stop codon as a result if abnormal splicing [94].

#### Diabetes obesity syndrome in CBA/CA mice

Male CBA/Ca mice have a spontaneous maturity onset diabetes obesity syndrome that occurs in a small proportion (10-20%). The incidence can increase upto 80% after inbreeding. It occurs at 12-16 weeks of age. Diabetic obesity syndrome is characterized by hyperphagia, obesity, hyperglycemia, hypertriglyceridemia and hyperinsulinemia. Exogenous insulin is resistant to mice. Female mice remains normal except for a slight increase in serum insulin [95].

#### Wellesley mouse

Jones described the Wellesley mouse. This mouse is a hybrid with predisposition to diabetes mellitus [96]. Like and Jones reported that the diabetic animals have elevated levels of immunoreactive insulin in serum, enlarged pancreatic islets and reduced insulin responsiveness in peripheral tissues [97].

### Chinese hamster

The occurrence of hereditary diabetes mellitus in the Chinese hamster (*Cricetulus griseus*) is described by Meier and Yerganian [98]. Diabetic hamsters have elevated blood sugar levels ranging from a normal of 110 mg% up to 600 mg%. Diabetic symptoms observed in Chinese hamsters are severe polyuria, glucosuria, ketonuria, and proteinuria. Administration of insulin, and oral antidiabetic drugs could be improved the diabetic symptoms. Histologically pathological changes are seen in sections of pancreas, liver and kidney. The numbers of pancreatic islets are decreased and the cells of the remaining islets are abnormal.

### Other species with inherited diabetic symptoms

#### Sand rat

The sand rat (*Psammomys obesus*) lives in the desert regions of North Africa and near east [99]. The animals develop diabetic symptoms when fed chow instead of an all vegetable diet. Within 2-3 months the diabetic syndrome in the sand rat usually develops. Severely hyperglycemic animals die prematurely from ketosis. Dubault et al recommend sand rat (*Psammomys obesus*) as a model of latent IDDM in NIDDM patients [100].

#### Spiny mouse

The spiny mouse (*Acomys cahirinus*) is found in the semi-desert areas of the Eastern Mediterraneann [101]. Diabetes occurs in about 15% of the animals under laboratory conditions. Diabetes is accompanied by hyperplasia of the endocrine pancreas. Some animals show obesity, mild hyperglycemia, and hyperinsulinemia and some have frank hyperglycemia with glucosuria that leads to fatal ketosis. All spiny mice characteristically have massive hyperplasia of pancreatic islets and increased pancreatic insulin content.

#### African hamster (*Mystromys Albicaudatus*)

Schmidt et al. described spontaneous diabetes mellitus in South African hamsters (*Mystromys albicaudatus*) [96]. Hyperglycemia, glucosuria, ketonuria, polyuria, polyphagia, polydipsia and pancreatic lesions include β-cell vacuolization, glycogen infiltration, nuclear pycnosis, margination of organelles, and β-cell death are the characteristics of the diabetic syndrome in this species.

#### TUCO-TUCO

Wise et al reported that the diabetic syndrome in Tuco-tucos (*Ctenomis talarum*) is similar to that in sand rats and spiny mice. However Tuco-tucos tend to have less hyperglycemia and are less prone to ketosis [102]. Many animals, mainly males, become hyperphagic. In few animals degranulation of β cell is the usual lesion in the pancreas, but amyloid hyalinization of islets has been observed.

#### MACACA NIGRA

Howard found a high incidence of spontaneous diabetes mellitus in *Macaca nigra *(Celebes black apes) [103]. Diabetes mellitus signs include hyperglycemia, decreased clearance of glucose, in intravenous tolerance tests, reduced insulin secretion and increased serum lipids. Atherosclerosis, thickened basement membranes of muscle capillaries, and cataracts are the secondary manifestations of diabetes mellitus [104].

### Transgenic Animals

#### Transgenic Mice

Transgenic mouse model of chronic hyperglycemia is described by Schaefer et al [105]. The extracellular ligand binding domain of the human insulin receptor can be expressed as a stable soluble protein that is efficiently secreted and binds insulin with high affinity. Expression under the mouse transferrin promoter of a transgene encoding a secreted derivative of the human insulin receptor in transgenic mice results in the accumulation of this high-affinity insulin-binding protein in the plasma. Alterations of glucose homeostasis are induced including postabsorptive hyperglycemia concomitant with increased hepatic glucose production and hyperinsulinemia. Palmiter et al. developed a method of deleting specific cell lineages that entailed microinjection into fertilized eggs of a chimeric gene in which a cellspecific enhancer/promoter is used to drive the expression of a toxic gene product [106]. Microinjection of a construct in which the elastase I promoter/enhancer is fused to a gene for diphtheria A polypeptide resulted in birth of mice lacking a normal pancreas because of the expression of the toxin in pancreatic acinar cells. A small pancreatic rudiment, containing islet and ductlike cells, was observed in some of the transgenic mice. Aichele et al. used a synthetic peptide corresponding to an immunodominant epitope of lymphocytic choriomeningitis virus glycoprotein (LCMV GP) to prime or to tolerize CD8+ T cells *in vivo *[107]. Peptidespecific tolerance was then examined in transgenic mice expressing LCMV GP in the β islet cells of the pancreas. These mice developed CD8+ T cell-mediated diabetes within 8-14 days after LCMV infection.

Specific peptide-induced tolerance prevented autoimmune destruction of β islet cells and diabetes in this transgenic mouse model. Oldstone et al. showed that virus infection triggers insulin-dependent diabetes mellitus in a transgenic mouse model [108]. Ablation of tolerance and induction of diabetes by virus infection in viral antigen transgenic mice was reported by Ohashi et al. [109]. Von Herrath et al. investigated how virus induces a rapid or slow onset insulin-dependent diabetes mellitus in two distinct transgenic mouse models. Von Herrath et al. evaluated the role of the costimulatory molecule B7-1 in overcoming peripheral ignorance in transgenic mice which expressed the glycoprotein or nucleoprotein of lymphocytic choriomeningitis virus as the self-antigen in pancreatic β-cells [110]. Von Herrath and Holz reported that pathological changes in the islet milieu precede infiltration of islets and destruction in β-cells by autoreactive lymphocytes in a transgenic model of virus-induced IDDM. RIP-LCMV transgenic mice that express the viral glycoprotein or nucleoprotein from lymphocytic choriomeningitis virus (LCMV) under control of the rat insulin promoter (RIP) in pancreatic β-cells develop autoimmune diabetes after infection with LCMV [111]. Upregulation of MHC class II molecules associated with the attraction/activation of antigen presenting cells to the islets occurs as soon as 2 days after LCMV inoculation of transgenic mice, clearly before CD4+ and CD8+ lymphocytes are found entering the cells. Moritani et al reported the prevention of adoptively transferred diabetes in non obese diabetic mice with IL-10-transduced islet-specific Th1 lymphocytes as a gene therapy model for autoimmune diabetes [112].

## Miscellaneous Models

### Invertebrate animal model

The invertebrate silk worm *Bombyx mori *is used for the identification of anti-diabetic drugs in this model. The silk worm fed a high-glucose diet (10% glucose containing diet) for 3 days. This increases 4 fold hemolymph sugar level compared with silk worms fed a normal diet. The hemolymph sugar level increased following intake of up to a 33% glucose diet without saturation. The other parameters like body size and weight was also influenced by this model.

The use of mammalian animals to screen for anti-diabetic drugs, however is not only very expensive from an animal husbandry perspective, but also prevents ethical problems in terms of animal welfare. It is far less costly to rear silkworms than mammals and a large number of larvae can be maintained in a small space [113].

### Diet Induced metabolic dysregulation

The model is clinically relevant for studying diet induced metabolic dysregulation. In this model non-human primates Baboon (*Papio hamadryas sp*.) is induced with high sugar, high fat diet after 12 hour fasting. The composition of diet includes 73% Purina Monkey Chow 5038 (a grain-based meal), 7% lard, 4% Crisco, 4% coconut oil, 10.5% flavored high fructose corn syrup, and 1.5% water. After 8 weeks of dietary exposure increase in body fat and triglyceride concentration is observed along with change in percentage glycosylated hemoglobin (HbA1c) and adipokines. The baboons and humans are genetically, anatomically, and physiologically very similar. Baboons and humans also appear to develop similar metabolic disorders, earlier studies have documented spontaneous obesity, insulin resistance, and a form of adult-onset diabetes in captive baboons [114].

## Conclusion

The methods cited in the above review have some advantages and disadvantages depicted in Table 1. However each and every newly synthesized drug goes into the market and used in human beings until and unless it studied on the laboratory animals. The characteristic features of disease and pathological changes during disease in small animals (rats or mice) are near about similar to that of human beings. Thus the preclinical study is always done before going to clinical study as human life is more precious than that of animals. The use of smaller animal models such as mice will also reduce the cost of experiments for test compound. Further, the selection of particular animal model is particularly depending on the investigator's choice whether to use inbred or outbreed, availability of particular strain, aim of scientific strategy, type of drug being sought, institutional financial and facility resources in the type 2 diabetes research and pharmaceutical drug discovery and development programme. The detailed investigations in these diabetic animal species are urgently required for better understanding of the disease mechanisms in much closely similar human situation as well as for discovering newer targets and drugs for the treatment of type 2 diabetes.

**Table 1:** Advantages and Disadvantages of different Models of Diabetes mellitus

S.No	Methods	Advantage	Disadvantage
**1(a)**	**Pancreatectomy in dogs**	Resembles human type 2 diabetes due to reduced islet beta cell mass Avoids cytotoxic effects of chemical diabetogens on other body organs	Involvement of cumbersome technical and post operative procedures Occurrence of some other digestive problems (as a result of part of excision of exocrine portion (deficiency of amylase enzyme) Dissection of alpha islets (glucagon secreting cells) too along with beta cells leading to problems in counter regulatory response to hypoglycemia Mortality is comparatively higher

**1(b)**	**Alloxan induced diabetes**	Selective loss of pancreatic beta cells leaving other pancreatic alpha and delta cells intact Residual insulin secretion makes the animals live long without insulin treatment Ketosis and resulting mortality is relatively less. Comparatively cheaper, easier to develop and maintain	Hyperglycemia develops primarily by direct cytotoxic action on the beta cells and insulin deficiency rather than consequence of insulin resistance Less stable and reversible because of the spontaneous regeneration of beta cells May produce toxic effects on other body organs Variability of results on development of hyperglycaemia is perhaps high

**1(c)**	**Streptozotocin induced diabetes**	Diabetes induced by streptozotocin is more stable This model can be used for longer experimental study	Comparatively costlier to develop Mortality is relatively more

**1(d)**	**Hormone induced diabetes**	Stable and irreversible diabetes can be induced Ketonuria and ketonemia occurs Not developed in small laboratory animals	Comparatively costlier to develop May affect other organs

**1(e)**	**Virus induced diabetes**	Stable and irreversible diabetes can be induced	Comparatively costlier to develop It develops Type 1 diabetes Handling of virus requires a technical expert

**1(f)**	**Other diabetogenic compounds**	Comparatively cheaper to develop Mortality is relatively less Can be developed in all type of experimental animals	May affect other organs Variability of results on development of hyperglycaemia is high

**1(g)**	**Insulin deficiency due to insulin antibodies**	State of insulin deficiency occurs Induce a transient diabetic syndrome	Comparatively costlier to develop Reversible at lower doses Ketonemia, ketonuria, glucosuria, and acidosis occurs in higher dose

**2(a)**	**Spontaneously diabetic rats**	Hyperglycemia persists for several days	A diabetes and obesity symptom overlaps Not identical to those in human disease

**2(b)**	**Spontaneously diabetic mice**	Hyperlipidemia can be studied as lipid contents increases	Polyphagia and polyuria occurs

**2(c)**	**Chinese hamsters**	Handling is comparatively difficult than rats and mice	Ketonuria, polyuria, polyphagia, polydipsia occurs

**2(d)**	**Other species with inherited diabetic symptoms**	Handling is comparatively difficult than rats and mice	Comparatively costlier to develop

**2(e)**	**Transgenic animals**	*In vivo *effect of single gene or mutation on diabetes can be studied Dissection of complex genetics of type 2 diabetes become easier	Costly method Experts are required

**3 (a)**	**Invertebrate animal model**	Lesser time is required No use of vertebrates Comparatively cheaper	The physiology and anatomy matches less with humans

**3 (b)**	**Diet Induced metabolic dysregulation**	Baboons and humans are genetically, anatomically, and physiologically very similar Cardiac complications can be studied	Handling of baboon is somewhat difficult Veterinarian is required Costly model

## Competing interests

The authors declare that they have no competing interests.

## Authors' contributions

SK Collected and arranged the data in sequence, drafted and correspond the manuscript. RS helped in collecting the data. NV checked and corrected the English language. SS helped to draft the manuscript. All authors read and approved the final manuscript.
